# Antibacterial activity of Betadine ( *Jatropha multifida* L.) stem extract on *Pseudomonas aeruginosa *growth *in* *vitro*

**DOI:** 10.12688/f1000research.123777.1

**Published:** 2022-10-27

**Authors:** Hendry Rusdy, Diah HI Damanik

**Affiliations:** 1 Department of Oral and Maxillofacial Surgery, Faculty of Dentistry Universitas Sumatera Utara, Medan, Sumatera Utara, 20155, Indonesia

**Keywords:** Betadine, sap, Jatropha multifida L., Pseudomonas aeruginosa L., Infection, Antibacterial, Nosocomial, Opportunistic.

## Abstract

**Background**: Today, people use plants to treat various types of diseases and improve human health. One of the medicinal plants is the Betadine plant ( *Jatropha multifida* L.). Betadine plants have many functions, especially the sap, leaves, fruit and seeds. The compound contents in Betadine stem sap, which is efficacious as an antimicrobial, are saponins, tannins, flavonoids and labaditin. One of the bacteria that cause infection is *Pseudomonas aeruginosa.* These bacteria can cause opportunistic and nosocomial infections.

**Methods**: This study was a true experimental laboratory with a post-test only control group design. This study used Betadine stem sap extract with concentrations of 25%, 50%, 75%, 100%, gentamicin cream 10% as positive control, and dimethyl sulfoxide (DMSO) solution as negative control. This study used the Kirby-Bauer diffusion method and the bacterium *Pseudomonas aeruginosa* was grown on nutrient agar media, then incubated for 24 hours and calculated using calipers. Research data were analyzed using one-way ANOVA test.

**Results**: The highest inhibition zone was group 50% (12.725 ± 0.2500 mm) while the lowest inhibition zone was group 100% (8.675 ± 0.5620 mm).

**Conclusions**: Betadine stem extract had antibacterial activity in inhibiting the growth of *Pseudomonas aeruginosa* bacteria, with the 50% concentration being the most effective in inhibiting the growth of *Pseudomonas aeruginosa* bacteria.

## Introduction

In Padang, West Sumatra Province, people use Betadine stem sap ( *Jatropha multifida* L.) to heal and eliminate external wound infections by applying Betadine stem sap on the injured body part. Indonesian people call this plant a Betadine plant because it has the same potential as an external wound medicine like Betadine in duration to heal an infected external wound. ^1^^–^^3^

According to Ivan *et al.* 2019, the Betadine plant can inhibit the growth of *Staphylococcus aureus* and *Pseudomonas aeruginosa.* ^4^ Betadine plant sap consists of multifidol, biobollein, multifidone, multifidil, flavonoid, labaditin, saponin and tannin. ^5^ In a study conducted by Aransiola *et al*. 2014 about the antibacterial and antifungal activity of Betadine plant sap, they found that the minimum inhibitory concentration (MIC) of Betadine plant sap gram negative bacteria ( *P. aeruginosa*, *E. coli*, and *Salmonella typhi*) was 66 mg/ml. These results indicate that the antibacterial activity found in the Betadine plant sap is lower in gram negative bacteria because gram negative bacteria are more resistant to antibacterials. ^6^

Based on WHO data in 2013, the mortality rate due to resistant bacterial infection is 700,000 people per year. ^7^ *P. aeruginosa* is an anaerobic, nosocomial and opportunistic bacterium. *P. aeruginosa* bacteria live in humid environments such as surgical instruments, 74% in dental water and dental disinfecting units used for more than 24 hours. *P. aeruginosa* was isolated in maxillary osteomilitis and brain abscesses in patients who had dental infections. This bacteria can cause meningitis, endocarditis, pneumonia and sepsis with an average mortality of 12–25%. This bacteria is resistant to many antibacterial including amoxicillin. Antimicrobial ingredients that are effective against *Pseudomonas aeruginosa* are hard to find, so natural resources such as Betadine plants can be used to suppress bacterial growth. ^5^^,^^8^^,^^9^

Based on the explanation above, the authors are interested in conducting research on “Antibacterial activity of Betadine stem ( *Jatropha Multifida* L.) extracts on *Pseudomonas aeruginosa* bacteria growth by *in vitro*”.

## Methods

### Study type

This study was a true experimental study with a post-test only control group design. True experimental study measured or observed data after the treatment was given.

### Location

Laboratory of Traditional Medicine and Microbiology, Faculty of Pharmacy, University of Sumatera Utara, Medan, Indonesia.

### Duration

Study duration was ± 2 month, from December 2019 to January 2020.

### Sample size

In calculating the size of experimental research samples, we used the Federer formula:

$$\left(t-1\right)\left(r-1\right)\geq 15$$

where t = number of treatment and r = number of repetition. The study used six treatment group: group 1 (25% Betadine stem sap extract), group 2 (50% Betadine stem sap extract), group 3 (75% Betadine stem sap extract), group 4 (100% Betadine stem sap extract), group 5 (gentamicin as a positive control), group 6 (dimethyl sulfoxide or DMSO as a negative control)

The number of repetitions (r) are four. So, 24 samples were obtained.

### Dependent variable

The dependent variable in this study was the sensitivity test growth of *Pseudomonas aeruginosa* that measured by the diameter of the inhibitory zone.

### Independent variable

The independent variable in this study was the concentration of Betadine stem sap extract used, which were 25%, 50%, 75% and 100%.

### Controlled variable

There were six controlled variable such as the growth media used for the bacterium *Pseudomonas aeruginosa* was nutrient broth agar, the incubation temperature of *Pseudomonas aeruginosa* is 37 ^o^C, the incubation time of *Pseudomonas aeruginosa* was 24 hours, the isolation and culture technique of *Pseudomonas aeruginosa* bacteria, sterilization of tools, the materials and media used, and operator skills.

### Uncontrolled variable

There were four uncontrolled variable such as the morphology of the Betadine stem, the growth time of the Betadine stem, the state of the soil and rainfall, the environment from which the Betadine plant originated, and the storage of the Betadine stem sap extract in the laboratory.

### Instruments

Autoclaves (Tomy ES 315, Japan), UV-VIS spectrophotometers (Orion Aquamate 8000, Germany), ovens (Memmert UN55, Germany), micropipets (Dragon Lab, China), Laminar air flow cabinet (Astec HLF I1200L), glass cuvettes, refrigerators (Thermo Scientific), incubators (Memmert, Germany), vortex mixers (Biosan, Latvia), glass tubes, glass petri dish, test tubes, inoculating loops, bunsen, measuring cups 20 ml, spatula, Vernier calipers (Electric Digital Capliper), analytical scales (Sartorious BSA323S-CW) and Erlenmeyer.

## Materials

Betadine stem sap extract 100% concentration ( *Jatropha multifida* L.), bacterial culture of *Pseudomonas aeruginosa* (ATCC ^®^ 140218, pure culture stock Faculty of Pharmacy, University of Sumatera Utara, Indonesia), DMSO solution, gentamicin ointment 10% (IKAGEN ^®^), aquadest, nutrient agar powder (Oxoid), nutrient broth agar powder (Oxoid), label paper, and 6 mm sterile paper discs.

### Procedures

First, all the instruments were sterilized with the autoclave at 121°C for 15 minutes and dried with an oven 170°C for 1–2 hours. Inoculating loop and pinset were sterilized with the bunsen flame. After that, took dilution of Betadine stem sap extract 100% with DMSO using a micropipet into a glass tube and measured with sartorious analytical scales (1 ml). The nutrient agar powder (28 g) was mixed with 1000 ml aquadest in an erlenmeyer flask with a spatula. This was heated for 2 hours at 100°C and after that saved in autoclave. Then, sterilized inoculating loops were used to take the isolated *P. aeruginosa* colonies and these were then placed into test tubes each containing the tilt media (contained 3 ml nutrient agar). The tube was put in an incubator at 37°C for 24 hours. Took *P. aeruginosa* colony using sterilized inoculating loops and than put into a tube that contain 10 ml nutrient broth agar. The tube was put on vortex mixers for 1 minute. Took 3 ml bacteria and put into glass cuvets, then put into UV-VIS spectrophotometers with 560 nm wavelength. The result must be 25% *transmittance.* In laminar airflow cabinet took 20 ml nutrient agar using measuring cups and 1 ml bacteria suspense using micropipets into glass petri dishes. The petri dishes were shaken slowly in a figure of eight movement until homogeneous. Paper discs were soaked with 25%, 50%, 75%, 100% Betadine stem sap extract, getntamicin and DMSO for 3 minutes. Then these were placed on the surface of the nutrient agar and a little pressure was applied. The petri dishes were labelled with label paper and placed in the refrigerator for 1 hour. Then the petri dishes were put into an incubator for 24 hours at 37°C. The diameter of inhibitory zone was clear zone in the petri dish. Measured it with caliper.

### Data analysis

Data was analyzed using software IBM SPSS Statistics Desktop 20.0 Windows Multilingual eAssembly (CRG2LML). The data was processed and analyzed with one way ANOVA test, and post hoc LSD (least significant difference).

## Results

**Figure 1.**
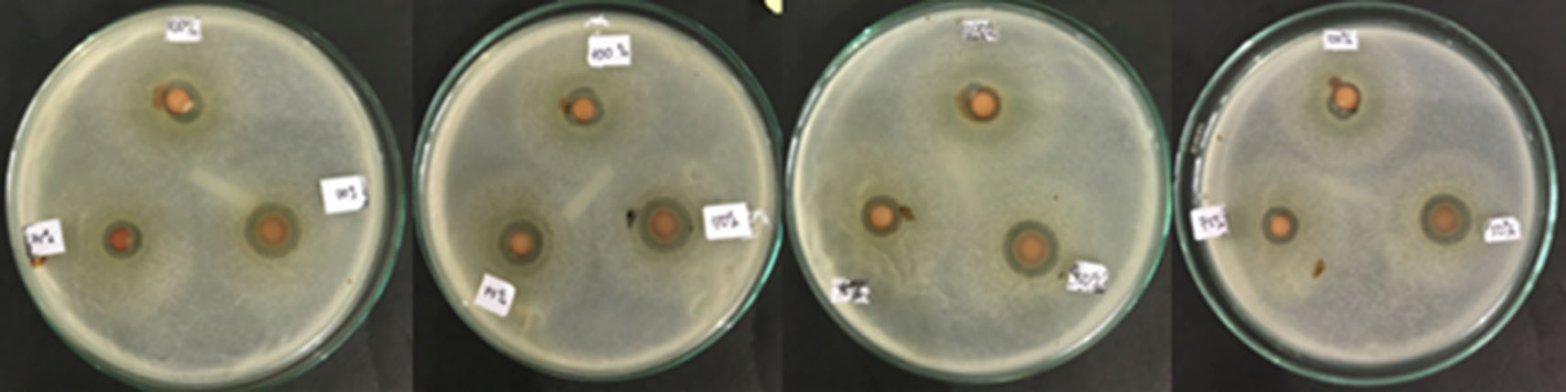
Inhibition zone group 100% (upper paper dics), group 75% (bottom left paper dics), group 50% (bottom right paper dics) with four repetitions.

**Figure 2.**
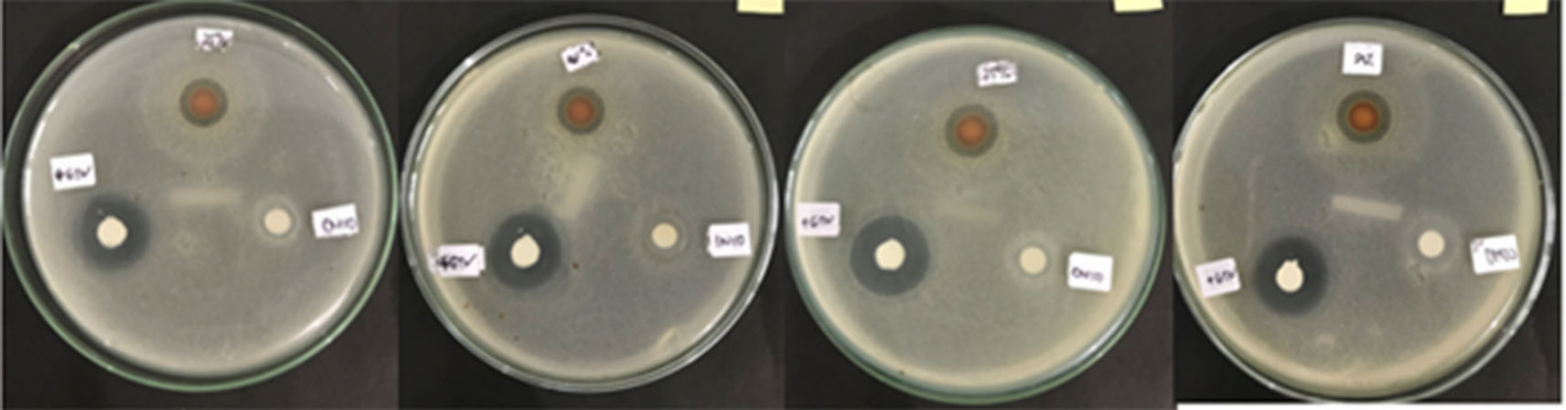
Inhibition zone group 25% (upper paper dics), group positive control (bottom left paper dics), group negative control (bottom paper dics) with four repetitions.

From this study, the lowest inhibition zone was produced by the Betadine stem sap extract group 100% (8.675 ± 0.5620 mm), while the highest inhibition zone was produced by the 50% (12.725 ± 0.2500 mm) Betadine stem sap extract group ( Figures 1 and 2). The Saphiro-Wilk test with a significance of p > 0.05 was used for the normality test and the data were found to be normally distributed (p > 0.05) ( Table 1). The data was distributed normally, so statistical analysis was continued using a one-way ANOVA analysis test and post hoc LSD to see whether there was a significant difference in the diameter of the inhibition zone between group 25%, 50%, 75%, 100%, K+, and K-. The ANOVA test showed a significant difference (p < 0.05) in the effect of the inhibition zone diameter between group 25%, 50%, 75%, 100%, K+ and K- ( Table 2). The LSD post hoc test was used to determine the significance of the inhibition of the growth of *Pseudomonas aeruginosa* between all groups in this study. The results of the LSD post hoc test are shown in Table 3.

**Table 1.** Results for measurement of inhibition zone diameter.

Repetitions	Inhibition Zone Diameter (mm)					
	100%	75%	50%	25%	K+	K-
1	8.2	8.6	12.4	11.9	17.8	-
2	9.3	9.4	12.7	12.1	17.9	-
3	9.0	9.1	12.8	12.5	18.6	-
4	8.2	8.6	13.0	12.6	18.8	-
**Mean**	**8.675**	**8.925**	**12.725**	**12.275**	**18.275**	**-**
**SD**	**0.5620**	**0.3948**	**0.2500**	**0.3304**	**0.4992**	**0.00**

**Table 2.** One-way ANOVA test results.

Treatment	Number of repetition	The average ± standard deviation	P-Value ANOVA
100%	4	8.675 ± 0.5620	p = 0.000
75%	4	8.925 ± 0.3948	
50%	4	12.725 ± 0.2500	
25%	4	12.275 ± 0.3304	
K+	4	18.275 ± 0.4992	
K-	4	-	

**Table 3.** LSD post hoc test results.

Konsentrasi	100%	75%	50%	25%	K+	K-
100%	-	0.371	0.000	0.000	0.000	0.000
75%	-	-	0.000	0.000	0.000	0.000
50%	-	-	-	0.116	0.000	0.000
25%	-	-	-	-	0.000	0.000
K+	-	-	-	-	-	0.000
K-	-	-	-	-	-	-

## Discussion

The study used Kirby-Bauer’s method. ^9^ This method is fast, easy, simple and produces effective results to show the antibacterial activity of a substance. ^9^ The solvent was DMSO which is able to dissolve polar and nonpolar compounds and does not have antibacterial activity so it does not change the results. ^10^ Factors that influence the diffusion of extracts into the media are the extent of the concentration gradient, solubility, diffused molecular mass, temperature and density of the solvent. ^6^

The group 25%, the inhibitory zone was 12.275 mm smaller than the group 50%, which was 18.275 mm. ^11^ This is because the group 50% had higher active ingredients than the group 25%. This is in line with previous research conducted by Brooks *et al.* ^3^ and Anggita *et al.* ^12^ in 2018 which states that the effectiveness of an antibacterial agent is influenced by the concentration of a given substance, the higher concentration has the higher active ingredient as an antibacterial, thus increasing the inhibition ability against microbes.

The inhibition zone group 75% the was 8.925 mm and group 100% was 8.675 mm. ^11^ The decrease in inhibition zone diameter due to the minimum diffusion power of the dense viscous extract is because it had a high solution density. The group 75% had minimum penetration power because the minimal amount of solvent. The group 100% had minimum penetration power because there was no solvent. Molecules moved slowly because it is more difficult to pass through denser media so it takes longer and results in smaller inhibitory zones. ^9^ This is similar to a study conducted by Komariah *et al.* 2013, where there was a decrease in antibacterial activity along with an increase in the concentration of the extract due to the higher concentration, the viscosity would increase so that the extract would be more difficult to diffuse into the agar media. ^13^

The antibacterial activity of the Betadine stem sap extract is related to its antibacterial content, namely phenols, tannins, saponins and labaditin. ^3^ Large molecule of phenol are able to activate essential enzymes in microbial cells even at low concentrations. ^14^

Phenol compounds contained in Betadine stem sap are flavonoids and tannins. ^10^ Flavonoids work by inhibiting nucleic acid synthesis, inhibiting cytoplasmic membrane function and inhibiting energy metabolism. Flavonoids can damage the outer membrane and cytoplasm of gram-negative bacteria, disturb the exchange of nutrient and metabolite and inhibit the energy supply for bacteria. ^15^^,^^16^

Tannins can cause lysis of bacterial cell membranes due to the differences in bacterial cells osmotic pressure. Tannins damage cell membranes by breaking the phosphate group H ^+^ so the phospholipid molecule breaks down into glycerol, carboxylic acid and phosphoric acid. Saponins are also contained in Betadine stem sap. Its mechanism of action is by interfering with bacterial cell membrane stability, causing bacterial cell lysis, damaging the cell membrane and releasing various important components of microbial cells, namely proteins, nucleic acids, nucleotides and others. ^15^^,^^16^ According to a study conducted by Barbosa *et al.* 2019, Betadine stem sap contains labaditin which is effective in gram-positive bacteria but less effective in gram-negative bacteria because of the more complex structure of gram-negative bacterial cell walls. ^17^

From the four group of Betadine stem sap extract that were tested, the strongest antibacterial activity found the group 25% and group 50%. ^11^ However, when compared with the positive control antibiotic gentamicin, Betadine stem sap extract was not more effective than gentamicin. This is due to the fact that many gram-negative bacteria contain lipids and little peptidoglycan. The outer membrane of the *Pseudomonas aeruginosa* is a bilayer that serves for the selective defense of compounds entering and exiting cells. The outer membrane consists of phospholipid (inner layer) and lipopolysaccharide (outer layer). This makes it difficult for active compounds to enter the cell so antibacterial activity of Betadine stem sap extract is smaller than gentamicin. ^17^^,^^18^

This research proves that Betadine ( *Jatropha multifida* L.) stem sap extract has antibacterial activity on inhibiting the growth of *Pseudomonas aeruginosa in vitro.* This result is the first step in the possibility of utilizing papaya leaf extract as an alternative natural ingredient for antibiotics in dentistry with the need for a series of tests such as clinical trials, toxicity and side effects so that this research can be utilized by the public.

## Conclusions

Based on the results, Betadine stem sap extract has an antibacterial activity on *Pseudomonas aeruginosa* growth *in vitro.* The most effective concentration to inhibit the growth of *Pseudomonas aeruginosa* is the group 50%, with the largest inhibitory zone diameter of 12.725 ± 0.2500 mm. This study has limitations, so further research is recommended to investigate using concentrations below 50% and using dilution methods to obtain the MIC (minimum inhibitory concentration) and MKC (minimum kill concentration).

## Data availability

### Underlying data

Figshare: Antibacterial Activity of Betadine (Jatropha multifida L.) Stem Extract on Pseudomonas aeruginosa Growth In-Vitro. https://doi.org/10.6084/m9.figshare.20359653. ^11^

This project contains the following underlying data:

• Data Inibition Zone of Each Repetition of Betadine Extract.csv (the file contains plain data of each concentration of Betadine stem sap extract with four repetitions)
• Test of Normality.csv (The file contains normal data distribution)
• Descriptives.csv (The file contains median, variance, standart deviation, minimum, maximum, range, interquartile range, skewness and kurtosis each concentration of Betadine stem sap extract).
• Case Processing Summary.csv (file contains of summary valid and missing antibacterial activity each consentration).
• Oneway Anova Test.csv (The file contains of data which is distinguish the antibacterial activity of each concentration of Betadine stem sap extract against Pseudomonas aeruginosa bacteria).
• Post Hoc Test.csv (The file contains of data which is show the significant difference between each concentration of Betadine stem sap extract).

Data are available under the terms of the Creative Commons Zero “No rights reserved” data waiver (CC0 1.0 Public domain dedication).
