# Supplementary material for: Antibacterial activity of Betadine ( Jatropha multifida L.) stem extract on Pseudomonas aeruginosa growth in vitro
Source: F1000Res. 2024 Apr 2;11:1222. Originally published 2022 Oct 27. [Version 2] doi: 10.12688/f1000research.123777.2 (PMC11106593; doi:10.12688/f1000research.123777.2)
Supplement: Peer review history

# Reviewer report: Reviewer response for version 2

**DOI:** 10.5256/f1000research.163442.r261892

**Published:** 2024-05-20

**Authors:** Siti Nur Hazwani Oslan

**Affiliations:** 1 Faculty of Food Science and Nutrition, Universiti Malaysia Sabah, Kota Kinabalu, Malaysia

The manuscript is scientifically sound and well-structured. The authors have presented their results effectively and responded comprehensively to previous comments.  The revisions have significantly improved the clarity and overall quality of the manuscript.

Is the work clearly and accurately presented and does it cite the current literature?

Partly

If applicable, is the statistical analysis and its interpretation appropriate?

Yes

Are all the source data underlying the results available to ensure full reproducibility?

Yes

Is the study design appropriate and is the work technically sound?

Yes

Are the conclusions drawn adequately supported by the results?

Yes

Are sufficient details of methods and analysis provided to allow replication by others?

Yes

Reviewer Expertise:

Microalgae; functional food; Food microbiology

I confirm that I have read this submission and believe that I have an appropriate level of expertise to confirm that it is of an acceptable scientific standard.

# Reviewer report: Reviewer response for version 2

**DOI:** 10.5256/f1000research.163442.r261893

**Published:** 2024-04-15

**Authors:** Khaga Raj Sharma

**Affiliations:** 1 Central Department of Chemistry, Tribhuvan University, Kirtipur, Central Development Region, Nepal

Now the paper seems ok and it can be accepted for indexing in the required format of the journal.

Is the work clearly and accurately presented and does it cite the current literature?

Yes

If applicable, is the statistical analysis and its interpretation appropriate?

Yes

Are all the source data underlying the results available to ensure full reproducibility?

Yes

Is the study design appropriate and is the work technically sound?

Yes

Are the conclusions drawn adequately supported by the results?

Yes

Are sufficient details of methods and analysis provided to allow replication by others?

Yes

Reviewer Expertise:

Natural products chemistry, extraction, isolation, characterisation of various natural products and to perform the biological activities, molecular docking, green synthesis of nanoparticles and comparison of biological activities.

I confirm that I have read this submission and believe that I have an appropriate level of expertise to confirm that it is of an acceptable scientific standard.

# Reviewer report: Reviewer response for version 1

**DOI:** 10.5256/f1000research.135915.r164184

**Published:** 2023-11-15

**Authors:** Siti Nur Hazwani Oslan

**Affiliations:** 1 Faculty of Food Science and Nutrition, Universiti Malaysia Sabah, Kota Kinabalu, Malaysia

Overall, the manuscript presents an interesting study on the antibacterial activity of Betadine stem (Jatropha Multifida L.) extracts on Pseudomonas aeruginosa bacteria growth by in vitro. The introduction provides good background information about using Betadine stem sap in West Sumatra Province to heal external wound infections and the potential of the Betadine plant as an external wound medicine. The methodology is clearly presented, with an appropriate sample size and controlled variables. However, the uncontrolled variables could be more clearly defined. The instruments and materials used are appropriate for the study. The results are presented in a clear and concise manner, with appropriate statistical analysis. However, the discussion section could be improved by providing a more in-depth interpretation of the results and how they relate to previous studies. Overall, the manuscript has potential, but some revisions are needed to strengthen the discussion and improve clarity.

Is the work clearly and accurately presented and does it cite the current literature?

Partly

If applicable, is the statistical analysis and its interpretation appropriate?

Yes

Are all the source data underlying the results available to ensure full reproducibility?

Yes

Is the study design appropriate and is the work technically sound?

Yes

Are the conclusions drawn adequately supported by the results?

Yes

Are sufficient details of methods and analysis provided to allow replication by others?

Yes

Reviewer Expertise:

Microalgae; functional food; Food microbiology

I confirm that I have read this submission and believe that I have an appropriate level of expertise to confirm that it is of an acceptable scientific standard, however I have significant reservations, as outlined above.

# Response

**Published:** 2024-03-25

**Authors:** Diah Harezky Irene Damanik

**Affiliations:** Faculty of dentistry sumatera utara universitas, Indonesia

Thank you for your suggestion and your positive comment. I appreciate it. As your suggestion we already update the new article version.

 We already more clearly defined the uncontrolled variables. We added more in-depth interpretation of the results to the discussion section and the relation to the previous studies.

# Reviewer report: Reviewer response for version 1

**DOI:** 10.5256/f1000research.135915.r173580

**Published:** 2023-06-05

**Authors:** Khaga Raj Sharma

**Affiliations:** 1 Central Department of Chemistry, Tribhuvan University, Kirtipur, Central Development Region, Nepal

I have gone through the manuscript titled **Antibacterial activity of Betadine ( *Jatropha multifida* L.) stem extract on *Pseodomonas aeruginosa *growth *in vitro*** and the followings are the queries and the suggestions

1. The preparation of plant extract or the sample preparation is not well mentioned.
2. It will be better to justify using DMSO of high concentration as the negative control while doing the antibacterial activity.
3. In the photographs of the Petri plates used to perform an antibacterial activity, the positive control, negative control, and the concentrations of the sample needed to be mentioned clearly by typing the text over the plates.
4. Better to write materials and methods instead of procedures.
5. Most of the activities performed in this study have been reported in the literature, it will be better to highlight the novelty of this research.
6. Identification of plant sample is missing. It is needed to mention the voucher specimen number of the plant. It will be clearer if the photograph of the plant is kept in the material and method section.
7. The paper is submitted to the Journal of Food Research, but the manuscript is related to medicinal plants and antibacterial activity. The manuscript seems not suitable for this journal. If the food values and using the plant in food properties makes relevant to this journal.

These are minor revisions and need to be incorporated in the revised manuscript for the acceptance of this paper.

Is the work clearly and accurately presented and does it cite the current literature?

Yes

If applicable, is the statistical analysis and its interpretation appropriate?

Yes

Are all the source data underlying the results available to ensure full reproducibility?

Yes

Is the study design appropriate and is the work technically sound?

Yes

Are the conclusions drawn adequately supported by the results?

Yes

Are sufficient details of methods and analysis provided to allow replication by others?

Yes

Reviewer Expertise:

Natural products chemistry, extraction, isolation, characterisation of various natural products and to perform the biological activities, molecular docking, green synthesis of nanoparticles and comparison of biological activities.

I confirm that I have read this submission and believe that I have an appropriate level of expertise to confirm that it is of an acceptable scientific standard, however I have significant reservations, as outlined above.

# Response

**Published:** 2024-03-07

**Authors:** Diah Harezky Irene Damanik

**Affiliations:** Faculty of dentistry sumatera utara universitas, Indonesia

Thank you for your suggestion. I appreciate it.

1. The new article version was clearly mention the preparation of the extract.
2. We used DMSO 100% as negative control.
3. We revised the petri plates figures by typing text over the plates.
4. We revised Procedures section with Material and Method
5. In the new article version, we added the novelty of this research.
6. In the new article version, we added Betadine plane photograph and vocer specimen number from new references.
7. This manuscript is related to Medical Plants Journal because it is explain about antibacterial activity.
